# *Klebsiella pneumoniae* Multiresistance Plasmid pMET1: Similarity with the *Yersinia pestis* Plasmid pCRY and Integrative Conjugative Elements

**DOI:** 10.1371/journal.pone.0001800

**Published:** 2008-03-19

**Authors:** Alfonso J. C. Soler Bistué, Daniel Birshan, Andrew P. Tomaras, Manisha Dandekar, Tung Tran, Jason Newmark, Duyen Bui, Nisha Gupta, Keziah Hernandez, Renee Sarno, Angeles Zorreguieta, Luis A. Actis, Marcelo E. Tolmasky

**Affiliations:** 1 Center for Applied Biotechnology Studies, Department of Biological Science, College of Natural Science and Mathematics, California State University Fullerton, Fullerton, California, United States of America; 2 Department of Microbiology, Miami University, Oxford, Ohio, United States of America; The Research Institute for Children, United States of America

## Abstract

**Background:**

Dissemination of antimicrobial resistance genes has become an important public health and biodefense threat. Plasmids are important contributors to the rapid acquisition of antibiotic resistance by pathogenic bacteria.

**Principal Findings:**

The nucleotide sequence of the *Klebsiella pneumoniae* multiresistance plasmid pMET1 comprises 41,723 bp and includes Tn*1331.2*, a transposon that carries the *bla*_TEM-1_ gene and a perfect duplication of a 3-kbp region including the *aac(6′)-Ib*, *aadA1*, and *bla*_OXA-9_ genes. The replication region of pMET1 has been identified. Replication is independent of DNA polymerase I, and the replication region is highly related to that of the cryptic *Yersinia pestis* 91001 plasmid pCRY. The potential partition region has the general organization known as the *parFG* locus. The self-transmissible pMET1 plasmid includes a type IV secretion system consisting of proteins that make up the mating pair formation complex (Mpf) and the DNA transfer (Dtr) system. The Mpf is highly related to those in the plasmid pCRY, the mobilizable high-pathogenicity island from *E. coli* ECOR31 (HPI_ECOR31_), which has been proposed to be an integrative conjugative element (ICE) progenitor of high-pathogenicity islands in other *Enterobacteriaceae* including *Yersinia* species, and ICE_Kp1_, an ICE found in a *K. pneumoniae* strain causing primary liver abscess. The Dtr MobB and MobC proteins are highly related to those of pCRY, but the endonuclease is related to that of plasmid pK245 and has no significant homology with the protein of similar function in pCRY. The region upstream of *mobB* includes the putative *oriT* and shares 90% identity with the same region in the HPI_ECOR31_.

**Conclusions:**

The comparative analyses of pMET1 with pCRY, HPI_ECOR31_, and ICE_Kp1 _show a very active rate of genetic exchanges between *Enterobacteriaceae* including *Yersinia* species, which represents a high public health and biodefense threat due to transfer of multiple resistance genes to pathogenic *Yersinia* strains.

## Introduction

Plasmids are important contributors to the rapid acquisition of antibiotic resistance by pathogenic bacteria through their ability to acquire resistance genetic determinants and to transfer them among bacteria belonging to the same or different genera and species [1], [2]. These resistance genes are usually located inside transposable elements, integrons, or ISCRs (insertion sequence common regions), which facilitate their mobility at the molecular level and at least in the case of transposons could help expanding plasmid's host range [1], [3], [4], [5]. In the past few years, considerable efforts have been made to completely sequence resistance plasmids and understand their biological properties [1].

*K. pneumoniae*, an important opportunistic pathogen, is the causative agent of community-acquired infections and more frequently of infections in the urinary tract and soft tissue, pneumonia, septicemia, and meningitis in hospitalized patients [6]. Hospital outbreaks caused by multiresistant *K. pneumoniae* strains have been described throughout the world [7], [8]. It has been proposed that Crohn's disease as well as ankylosing spondylitis are linked to *Klebsiella* infection possibly through the mechanism of molecular mimicry [9]. *K. pneumoniae* has also been identified as the causative agent of liver abscess [10]. However, *K. pneumoniae* infections are especially dangerous in neonatal wards [6]. With the rise in the number of strains harboring resistance genes, usually plasmid-borne, the mortality of *K. pneumoniae* infections has increased and treatment has become more complicated [11], [12], [13]. A multiresistant *K. pneumoniae* isolate that caused high mortality in a neonatal ward harbors the self-transmissible plasmid pMET1, which carries the genetic determinants for resistance to several aminoglycosides and β-lactams [12]. In this paper we report the complete sequence and analysis of pMET1, and a comparative study with the *Yersinia pestis* cryptic plasmid pCRY, the mobilizable high-pathogenicity island found in *Escherichia coli* strain ECOR31 (HPI_ECOR31_), an integrative conjugative element (ICE) that is a possible progenitor of high-pathogenicity islands in other *Enterobacteriaceae* including *Yersinia* species, and an ICE (ICE_Kp1_) present in a clinical *K. pneumoniae* isolate. Our results indicate that pMET1 and pCRY are closely related and most possibly can be mobilized between *Yersinia* and other *Enterobateriaceae*, and be stably maintained in these bacteria. Furthermore the presence of elements of these plasmids in HPI_ECOR31_ and at least another ICE is an indication of a very active genetic exchange between these bacteria, which represents a high public health and biodefense threat due to transfer of multiple resistance genes to pathogenic *Yersinia* strains.

## Results and Discussion

**Figure 1:**
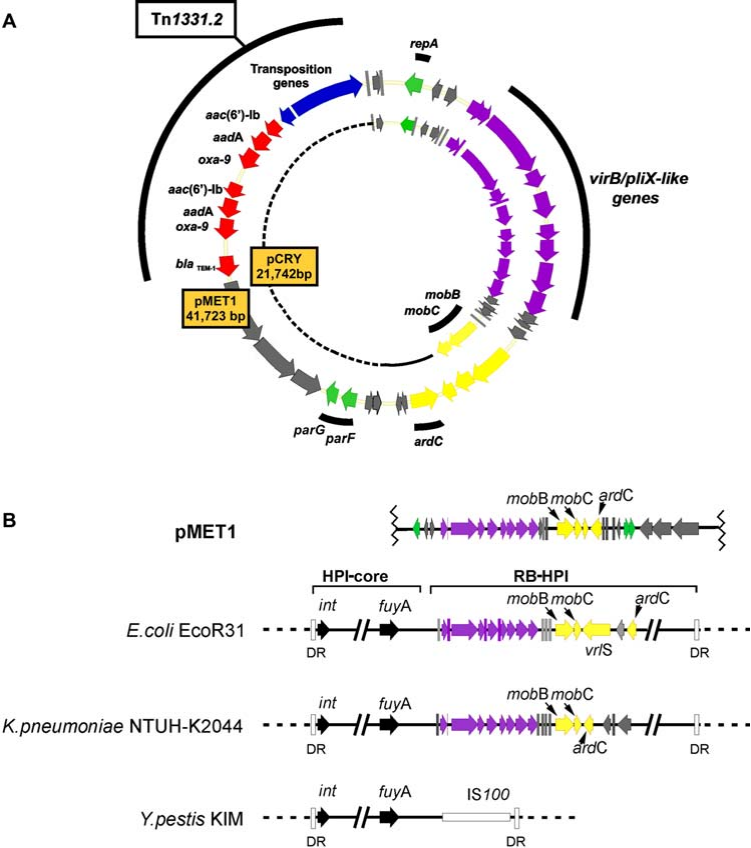
Genetic map of pMET1 and comparison to plasmid pCRY and chromosomal elements. A. The genetic maps of pMET1 and pCRY are compared showing the homologous regions. The arrows indicate genes locations and orientation. Genes with different functions are shown with different colors and if the genes in the different structures shown are homologus they are represented with the same colors. Yellow: mobilization; green: replication and partition; red: antibiotic resistance; purple: virB/pilX-like; blue: transposition; grey: unknown. Since pCRY is smaller than pMET1, to represent it in circular form a dotted line was added to fill the gap. Solid line represents non-homologous DNA. B. Comparison of a pMET1 region with chromosomal HPIs or ICEs is shown using a linearized version of the plasmid. The HPIs shown are those from *E. coli* ECOR31 (HPI_ECOR31_) [43], *K. pneumoniae* NTUH-K2044 (ICE_Kp1_) [44], and *Y. pestis* KIM (HPI_Yp_)[43]. The diagram shows the HP core regions, which are not at scale and are represented as in [43], and the RB-HPIs. The sequence described in this manuscript has been deposited in GenBank, accession number is EU383016.

The 41,723 bp multiresistance plasmid pMET1 (GenBank accession number EU383016) includes the replication region, a type 4 secretion system (T4SS), a partition system, and a transposable element that includes two genes coding for aminoglycoside modifying enzymes and two β-lactamases. Table 1 and Fig. 1 summarize the characteristics of pMET1 including the predicted functions of the ORFs on the basis of amino acid sequence comparison.

**Table 1:** Summary of ORFs found in pMET1.

ORF‡	Position	G+C Content (%)	Protein (aa)	Best Blast-X Hit	*E* value	Identity/similarity	Accession No.
1	1,387–674	45	238	Putative RepA protein [pCRY, *Yersinia pestis* biovar Microtus str. 91001]	2e-119	89/95	NP_995415
2	2,198–1,839	42.6	118	Putative transcriptional regulator [pCRY, *Yersinia pestis* biovar Microtus str. 91001]	2e-42	72/84	NP_995417
3	2,451–2,906	48.7	151	Transcription antiterminator [pCRY, *Yersinia pestis* biovar Microtus str. 91001]	2e-83	99/100	NP_995418
4	3,534–4,244	57.3	236	Type IV secretory pathway VirB1 component [pCRY, *Yersinia pestis* biovar Microtus str. 91001]	2e-132	99/99	NP_995421
5	4,241–4,546	56.2	101	Type IV secretory pathway VirB2 component [pCRY, *Yersinia pestis* biovar Microtus str. 91001]	9e-31	98/99	NP_995422
6	4,559–7,297	52.8	912	Pilx3-4/VirB3-4-like protein [*Escherichia coli*]	0.0	99/100	AAP70302
7	7,316–8,026	49.2	236	Type IV secretion system VirB5 component [pCRY, *Yersinia pestis* biovar Microtus str. 91001]	2e-98	84/90	NP_995424
8	8,275–9,351	46.4	358	Type IV secretory pathway VirB6 component [pCRY, *Yersinia pestis* biovar Microtus str. 91001]	1e-155	87/95	NP_995426
9 †	9443–9580	57.4	45	Hypotetical protein[pCRY, Yersinia pestis biovar Microtus str. 91001]	9e-13	91/91	AE017044.1
10	9,620–10,255	55.4	211	Type IV secretion system VirB8 component [pCRY, *Yersinia pestis* biovar Microtus str. 91001]	4e-105	99/100	NP_995427
11	10,252–11,160	56.9	302	Type IV secretory pathway VirB9 component [pCRY, *Yersinia pestis* biovar Microtus str. 91001]	1e-170	99/100	NP_995428
12	11,201–12,475	57.4	424	Type IV secretory pathway VirB10 component [pCRY, *Yersinia pestis* biovarMicrotus str. 91001]	0.0	96/97	NP_995429
13	12,465–13,490	47.6	341	Type IV secretory pathway VirB11 component [pCRY, *Yersinia pestis* biovar Microtus str. 91001].	0.0	99/100	NP_995430
14	13,487–13,885	46.5	132	Hypothetical protein YP_pCRY17 [pCRY, *Yersinia pestis* biovar Microtus str. 91001]	8e-73	98/100	NP_995431
15	13,921–14,226	42.4	101	Hypothetical protein YP_pCRY18 [pCRY, *Yersinia pestis* biovar Microtus str. 91001]	3e-52	99/99	NP_995432
16	14,251–14,565	40	104	Putative dopa decarboxylase protein remnant [*Yersinia pestis* biovar Microtus str. 91001]	8e-41	96/97	NP_995433
17	15,331–17,082	39.8	583	Putative mobilization MobB protein [pCRY, *Yersinia pestis* biovar Microtus str. 91001]	0.0	99/99	NP_995436
18	17,079 –17,846	48.7	255	Putative mobilization protein MobC [pCRY, *Yersinia pestis* biovar Microtus str. 91001]	9e-144	99/100	NP_995437
19	17,949–18,749	54.2	168	Putative endonuclease [pK245, *Klebsiella pneumoniae*]	2e-54	62/76	ABG56794
20	19,631–18,663	53.3	320	Antirestriction protein ArdC [p29930, *Yersinia enterocolitica*]	3e-152	86/92	CAD58578.
21	20,016–20,351	33.7	112	Hypotetical protein AmetDRAFT_2972 [*Alkaliphilus metalliredigenes* QYMF]	2e-14	39/86	ZP_00799958
22	20,348–20,680	31.1	111	Hypothetical protein AmetDRAFT_2972 [*Alkaliphilus metalliredigenes* QYMF]	2e-05	43/60	ZP_00799958
23	21,353–21,060	45.8	99	Transcriptional regulador [*Escherichia coli*]	5e-34	72/86	CAH19146
24	21,706–21,356	47.8	117	Hypothetical protein [*Escherichia coli*]	1e-43	71/84	CAH19145
25	22,100–22,717	45.8	205	Plasmid partitioning protein ParF [p29930, *Yersinia enterocolitica*]	3e-84	77/90	CAD58556
26	22,772–23,011	47.3	79	Partitioning protein ParG[p29930, *Yersinia enterocolitica*]	9e-16	53/70	CAD58557
27	24,886–23,630	46.8	417	Hypothetical protein V12B01_09786 [*Vibrio splendidus* 12B01]	6e-134	59/74	ZP_00991171
28	26,905–24,890	46.6	675	Hypothetical protein V12B01_09791 [*Vibrio splendidus* 12B01]	0.0	73/84	ZP_00991172
29	29,632–26,948	45.4	892	Hypothetical protein V12B01_09796 [*Vibrio splendidus* 12B01]	0.0	67/80	ZP_00991173
30	30,731–29,871	50.3	286	Beta-lactamase precursor (TEM-1)* [pJHCMW1, Tn*1331*, *Klebsiella pneumoniae*]	2e-152	100/100	NP_608310
31	32,270–31,431	50.3	279	Beta-lactamase precursor * (Penicillinase) (Oxacillinase) (OXA-9)	1e-157	100/100	NP_608309
32	33,103–32,315	53.6	262	Adenylyltranferase [pJHCMW1, *Tn1331*, *Klebsiella pneumoniae*]*	6e-145	99/100	NP_608308
33	33,778–33,173	54.1	201	Aminoglycoside 6′-N-acetyltransferase type Ib (AAC(6′)-Ib) * [pJHCMW1, Tn*1331*, *Klebsiella pneumoniae*]	1e-112	100/100	NP_608307
34	35,317–34,478	50.3	279	Beta-lactamase precursor * (Oxacillinase, OXA-9) [pJHCMW1, *Tn1331*, *Klebsiella pneumoniae*]	1e-157	100/100	NP_608309
35	36,150–35,362	53.6	262	Adenylyltranferase [pJHCMW1, *Tn1331*, *Klebsiella pneumoniae*]*	6e-145	99/100	NP_608308
36	36,825–36,220	54.1	201	Aminoglycoside 6′-N-acetyltransferase type Ib (AAC(6′)-Ib) * [pJHCMW1, Tn*1331*, *Klebsiella pneumoniae*]	1e-112	100/100	NP_608307
37	37,565–37,008	54.3	185	Resolvase [pJHCMW1, *Tn1331*, *Klebsiella pneumoniae*] *	2e-95	99/100	NP_608306
38	37,694–40,732	51.8	1015	Transposase [pJHCMW1, *Tn1331*, *Klebsiella pneumoniae*] *	0.0	100/100	NP_608305
39	41,223–40,942	52.7	93	Predicted transcriptional regulator [pCRY, *Yersinia pestis* biovar Microtus str. 91001]	7e-37	100/100	NP_995443
40	41,157–41,516	52.1	120	Hypothetical protein YP_pCRY30 [pCRY, *Yersinia pestis* biovar Microtus str. 91001]	1e-50	97/98	NP_995444
41	41,545–41,204	53	107	Hypothetical protein plu0442 [*Photorhabdus luminescens* subsp.laumondii TTO1]	2e-27	60/73	NP_927795

* Best hit selected according to genetic coherence rather than a minimal difference in score.

† Done with tblastn

‡ Including stop codon

### The transposable element Tn*1331.2*

**Figure 2:**
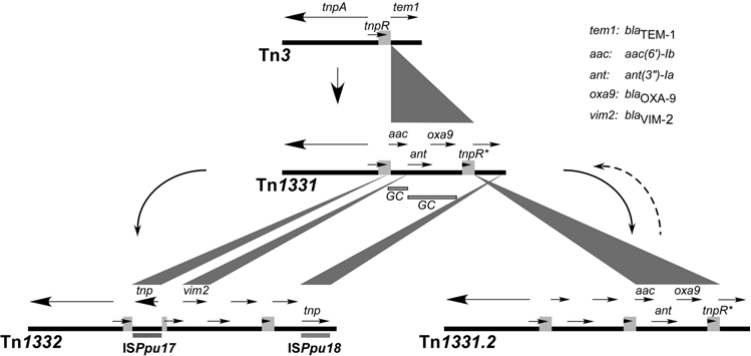
Genetic organization of transposons Tn*3*, Tn*1331*, Tn*1332*, and Tn*1331.2.* The horizontal arrows indicate the location and direction of transcription of the genes. The genes are named only once for the sake of clarity. The light gray blocks show the 520-bp region direct repeats. Note that in Tn*1332* one of the direct repeats is interrupted by an insertion of IS*Ppu17*. The upper vertices of the dark gray triangles show points of insertion of DNA fragments that generate a new transposable element. The length of the base of each triangle defines the sizes of such DNA insertions. The curved dashed arrow to the right indicates that the transition Tn*1331.2* to Tn*1331* by deletion of one of the 3,047-bp occurred in the laboratory but it has not been confirmed to occur in nature. Stippled bar below Tn*1331* indicates potential gene cassettes (GC).

The plasmid pMET1 includes an 11,042-bp transposon, named Tn*1331.*2 [12] that is highly related to Tn*1331* (see Fig. 2) [14], [15]. Tn*1331* can be considered an evolutionary product of Tn*3* by the addition of a 3,047-bp DNA region, which has the structure of the variable portion of the integrons, flanked by 520-bp direct repeats (Fig. 2). This region includes three resistance genes with a structure consisting of *aac(6′)-Ib-attC-aadA1-bla*_OXA-9_ *-attC*, potentially defining two gene cassettes including *aac(6′)-Ib-attC* and *aadA1-bla*_OXA-9_ *-attC* [14], [16], [17]. As a result of the presence of these three genes and *bla*_TEM_, cells harboring Tn*1331* are resistant to several aminoglycosides and β-lactams. Possible mechanisms for the generation of Tn*1331* have been discussed previously [16], [18]. Tn*1331.2* has a perfect duplication of the 3,047-bp DNA region and as a consequence a 520-bp including a C-terminal fragment of *tnpR* is found as three direct repeats flanking the potential gene cassettes (Fig. 2). As it is the case for Tn*1331* [14], the Tn*1331.2 tnpA* differs from the Tn*3* version of the gene in the 9-nucleotide repeats that in Tn*3* code for the amino acid sequence GFHGFH. Tn*1331.2* includes only one copy of this 9-bp fragment coding for the amino acid sequence GFH.

The duplication of the 3,047-bp DNA fragment including the resistance genes could be a strategy to increase the gene dosage when the transposon is present in a low copy number plasmid. Mechanisms of gene duplication when they are flanked by direct repeats have been described before [19], [20], [21]. Although we have observed a transition from Tn*1331.2* to Tn*1331* by the loss of one of the 3,047-bp repeats in the laboratory, we do not know if this is a process that happens in nature (dashed arrow, Fig. 2). While for many years no evolutionary derivatives of Tn*1331* other than the duplication to render Tn*1331.2* had been isolated, there was a recent report of such a phenomenon. The transposon Tn*1332*, isolated from *Pseudomonas putida*, can be considered as Tn*1331* with the insertion of three DNA fragments that include the metallo-ß-lactamase gene *bla*_VIM-2_, and the insertion sequences IS*Ppu17* and IS*Ppu18*, respectively (Fig. 2) [22].

### Replication and partition

**Figure 3:**
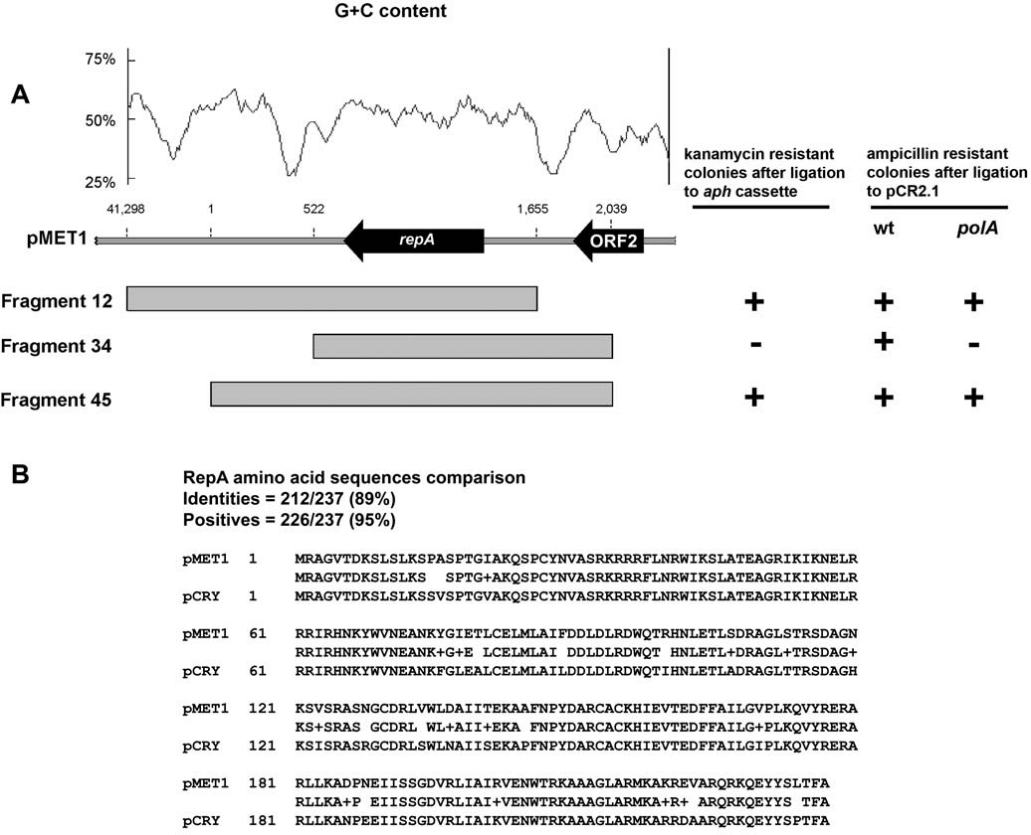
Replication region of pMET1. A. The bar shows a genetic map of the pMET1 replication region and the GC content plot generated using a window size of 100 bp on top. Recombinant clones were obtained by inserting the indicated fragments into pCR2.1 or ligated to the pUC4K *aph* cassette. The ability to be maintained in *E. coli* C2110 (a *polA* mutant) of the recombinant plasmids made using pCR2.1 as vector is indicated to the right by a + or − sign. The ability to generate kanamycin resistant colonies in *E. coli* TOP10 of the indicated fragments when ligated to the *aph* cassette from pUC4K is also represented by a + or − sign. B. BLASTP comparison of the amino acid sequences of the putative RepA proteins from pMET1 and pCRY.

Blastn analysis revealed a pMET1 region of high homology to the *Y. pestis* 91001 plasmid pCRY putative replication region [23] (schematically shown in Fig. 1A). The characteristics of this region include two AT-rich DNA segments flanking ORF1, named RepA, whose amino acid sequence showed 89% identity and 95% similarity to the pCRY RepA putative protein (Fig. 3A and B). Lower but still significant similarities were found to other replication proteins from plasmids isolated from *Buchnera aphidicola* [24], [25] and *Y. enterocolitica* plasmid pYVe227 [26]. To functionally identify the replication region of pMET1, three fragments 12, 34, and 45 (coordinates 41298-1655, 522-2039, and 1-2039 respectively)(Fig. 3A) were ligated to the kanamycin resistance fragment and used to transform *E. coli* TOP10. Kanamycin resistant colonies were obtained only when the recombinant clones included the fragments 12 or 45 (Fig. 3A). These results were confirmed by cloning all three fragments using pCR2.1 as vector, and attempting to transform the DNA polymerase I deficient *E. coli* C2110. While all three recombinant plasmids replicated in a wild-type *E. coli* strain, only recombinant clones including fragment 12 or 45 could be stably maintained in *E. coli* C2110. The recombinant clone harboring the fragment 34 was unable to replicate in the absence of DNA polymerase I (Fig. 3A). Therefore the results of both experiments defined a replication region encompassing nucleotides 1 to 1655, which includes two essential features: one complete ORF, named *repA*, and the AT-rich sequence between nucleotides 1 and 522 (Fig. 3A). This latter region likely includes the *ori* locus. These two features are conserved in the pCRY replication region: the AT rich sequence is conserved as the G+C content profile is almost identical to pMET1, and *repA* shows 95% similarity and 89% identity to pMET1 RepA when translated (Fig. 3B). On the other hand, the overall identity at the nucleotide level between pMET1 and pCRY for this locus is 89%. This high relatedness between the pMET1 and pCRY replication regions strongly suggests that pMET1 can be replicated in *Yersinia* species.

**Figure 4:**
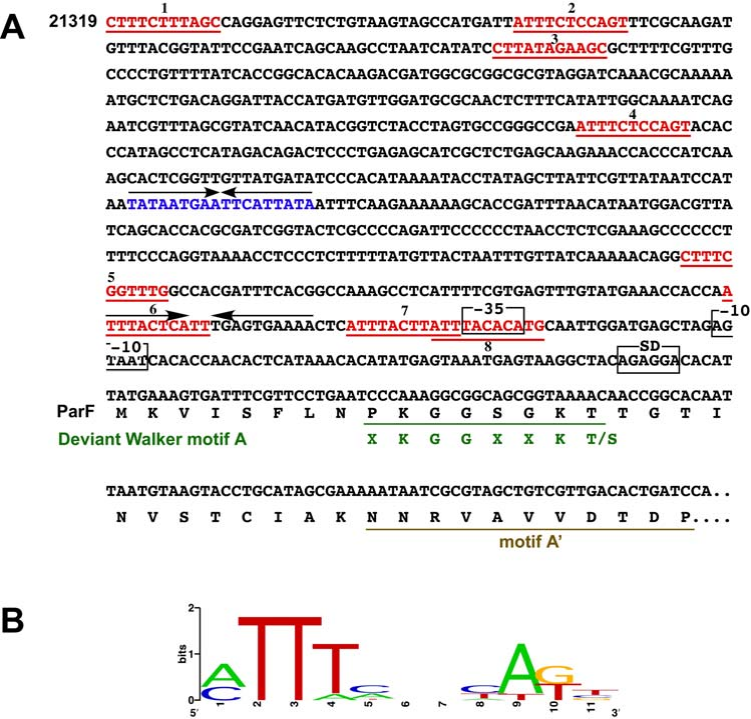
Genetic structures located upstream of *parF* and *parG.* A. The direct repeats within the pMET1 putative *parH*-like locus are shown in red. The diagram also shows the −35 and −10 sequences, as well as the inverted repeats (arrows). The inverted repeat within the putative *parH* locus is shown in blue. The beginning of the ParF amino acid sequence including the deviant Walker motif A and motif A' are shown. B. Logo plot [60], [61] of a multiple alignment of the direct repeats shown in red.

Plasmid pMET1 includes a putative partition region that has the general organization of that of plasmid pTP228, also known as the *parFG* locus [27]. The pMET1 partition region encompasses nucleotides 22,100 to 23,011 and includes two ORFs (ORF25 and ORF26, Table 1) encoding two putative proteins with strong homologies to ParF and ParG. The ParF homolog is a 205-amino acids protein that has shown significant homology with numerous proteins that belong to the type Ib subgroup of the ParA superfamily that is related to the MinD subgroup of cell division proteins [28], [29], [30]. These proteins are Walker-type ATPases that include a deviant motif A, which contains a conserved ‘signature’ lysine (XKGGXXKT) [29], [31], [32]. This motif as well as motif A' [29] are shown in Fig. 4A. The maximum pMET1 ParF identities and similarities were found with those encoded by the cryptic *Y. enterocolitica* plasmid p29930 [33] (90% similarity and 77% identity), the multidrug resistance plasmid pTP228 from *Salmonella enterica* subsp. *Enterica* serovar Newport [27] (87% similarity and 72% identity), and the *Erwinia amylovora* Ea88 plasmid pEA29 [34] (85% similarity and 73% identity).

Unlike with ParF, the pMET1 ParG has significant homologues only in p29930 (70% similarity and 53% identity), pTP228 (71% similarity and 58% identity), and pEA29 (68% similarity and 51% identity), generally known as ParB-like proteins. Studies on the pTP228 ParG protein showed that it acts as a dimer, may interact in a sequence-specific manner with a direct repeats-containing DNA region located upstream of *parF* known as *parH*, stimulates ATP hydrolysis by ParF, and modulates polymerization of ParF [35]. In addition, the ParG dimer binds an operator and acts as an autoregulator of the *parFG* operon [31]. Structures of parB-like proteins alone or in complex with DNA have been determined [36], [37], [38]. Other plasmids that have ParF homologues include proteins that are functional analogs but heterologs to ParG. The nucleoprotein complex has been recently called “segrosome” and is essential to ensure proper partition of the plasmid molecules [31]. Analysis of the pMET1 region upstream of the *parF* and *parG* genes showed an organization similar to that found in pTP228, which includes the *parH* locus and a putative operator (Fig. 4A)[31], [39]. Interestingly, although the ParG proteins from pTP228 and pMET1 share homology, the repeats found upstream of *parF* in pMET1 are unrelated to those in pTP228. This region includes eight imperfect direct repeats that stretch over the promoter region. A graphical representation (sequence logo) of the multiple alignment of these repeats is shown in Fig. 4B. A region that includes some or all of these direct repeats could play the role of *parH* in pMET1 partition. Fig. 4A also shows the putative promoter, the −35 sequence is located within two partially overlapping direct repeats and upstream of this structure the repeat number 6 is part of an inverted repeat. One or more of these structures could function as operators, binding of ParG to one or both of these two structures may result in inhibition of transcription of *parF* and *parG*. Within the putative *parH* region there is also a perfect inverted repeat (Fig. 4A) unrelated to the putative ParG binding sites. The function of this structure is unknown.

### Conjugation

Type 4 secretion systems (T4SSs) encoded by plasmids are involved in bacterial conjugation and transport of effectors to eukaryotic cells during infection [40]. Bacterial conjugation plays an important role in dissemination of antibiotic resistance genes at the cellular level [41], [42]. Previous studies indicated that pMET1 is self-transmissible [12], analysis of its nucleotide sequence shows that the transfer region of pMET1 lies within a ca. 16-kbp DNA segment, the ATG of the first gene, *virB1*, occurs at coordinate 3534 and the ATG of the last gene, *ardC* (which is transcribed opposite to all the rest) at coordinate 19,631. A block of predicted coding products (named *VirB1*–*VirB11*) are highly homologous to proteins that make up the mating pair formation complex (Mpf) in a large number of plasmids and other DNA elements with the highest identity with those in the *Y. pestis* 91001 plasmid pCRY [23], the high pathogenicity island HPI_ECOR31_ [43], and ICE_Kp1_, an ICE found in the primary liver abscess causing *K. pneumoniae* NTUH-K2044 [44] (see Fig 1B, Table 1, and Table 2). The putative functions of these proteins are discussed in several reviews [40], [42], [45]. In all four cases the *virB3* and *virB4* genes are fused. An homolog of published VirB7 proteins is absent in all four strucutures. However, a 45-amino acids hypothetical protein (ORF9) is present between *virB6* and *virB8* in all four elements. This high conservation and the synteny suggest that this ORF might play a role equivalent to that of VirB7. The pMET1 *vir-*like genes are followed by ORFs of unknown function and the predicted DNA transfer (Dtr) group of genes that potentially code for MobB, MobC, and an endonuclease. While the pMET1 MobB and MobC showed 99% identity to those in the *Y. pestis* plasmid pCRY, the endonuclease showed highest identity with that of the *K. pneumoniae* plasmid pK245, which carries genes coding for resistance to quinolones and extended-spectrum β-lactamases [46], and the cryptic *Y. enterocolitica* plasmid p29930 [33]. Interestingly, no significant homology was found with the nuclease encoded by pCRY. Further analysis of the pMET1 mobilization region showed a structure *oriT-mobB-mobC*, similar to those in other elements such as the mobilizable HPI_ECOR31_ [43] or the plasmid CloDF13 [47]. The pMET1 region upstream of *mobB* has 90% identity with that of the HPI_ECOR31_. As it is the case in this element, there are two *nic* (or *oriT*) sites in pMET1 due to their location inside inverted repeat 2 (IR2). It is then possible that either strand of pMET1 can be transferred to the recipient cell, an unusual phenomenon [48]. Furthermore, studies on the plasmid R1162, which also possesses two *oriT*s, suggested that transfer can be initiated at one of them and terminated at the other with the inverted repeat playing a role in termination [49].

**Table 2:** Comparison of amino acid sequences of VirB/PliX-like proteins

	pCRY AE017044.1	ICE_ECOR31_ AY233333.1	ICE_Kp1_ AB298504.1
VirB1	99/99	63/77	63/77
VirB2	98/99	76/85	76/85
VirB3-4-like	97/98	99/100	98/99
VirB5	84/90	83/98	84/89
VirB6	87/95	87/92	95/96
ORF9	97/97	100/100	97/100
VirB8	99/100	98/100	99/99
VirB9	98/100	99/99	98/99
VirB10	96/97	95/96	95/95
VirB11	99/100	99/100	97/99

Values indicate identity/similarity obtained by blastx comparison of each pMET1 ORF and the corresponding hypothetical protein from pCRY, ICE_ECOR31_ and ICE_Kp1_.

Downstream of the endonuclease gene, there is an ORF that is transcribed in the opposite direction to all the genes described above with homology to the p29930 *ardC* [33], which codes for a putative antirestriction protein. The pSA ArdC protein has been studied in some detail. It has been shown to bind ssDNA and to protect single-stranded but not double-stranded plasmid DNA *in vitro* against the activity of *Hha*I, an enzyme that cleaves both single- and double-stranded DNA. It was proposed that ArdC is transported to the recipient cell with the transferred single-stranded DNA and protects the incoming single-stranded DNA from the host endonucleases [50].

### Comparative analysis of pMET1 and the *Y. pestis* pCRY plasmid

pCRY is a 21,742-bp cryptic plasmid recently isolated from *Y. pestis* strain 91001 [23]. Comparative analysis of pMET1 and pCRY DNA sequences revealed highly similar backbones (see Fig. 1A). These analyses showed that the ca. 18-kb fragment including the replication region and the T4SS involved in conjugation of pMET1 shares high sequence homology as well as gene synteny with that of pCRY. These results suggest that they have a common ancestor and it would not be surprising if other plasmids with the same backbone but carrying other mobile genetic elements including resistance genes were found in other *Enterobacteriaceae* isolates. In the particular case of *Y. pestis*, our findings, taken together with the recent description of other multiresistance plasmids of *Yersinia* that share their backbone with plasmids of *Salmonella* [51], and the finding of a common ancestry between the *Y. pestis* pMT1/pFra plasmid and a cryptic plasmid from a clinical isolate of *S. enterica* serovar Typhi [52] indicate that there must be an active transfer of plasmids between *Yersinia* and other *Enterobacteriaceae* by direct contact or through other intermediate bacteria resulting in a high risk of dissemination of antibiotic resistance genes to pathogenic *Y. pestis* strains.

### Comparative analysis of pMET1 and the integrative and conjugative element of *E. coli* ECOR31

The pMET1 T4SS is related to those found in the ICE_Kp1_ and HPI_ECOR31_ (Fig. 1B). A study on HPI_ECOR31_ showed that it shares common structures, such as the genes for synthesis of the siderophore yersiniabactin, with other high-pathogenicity islands found in *Enterobacteriaceae* including *Yersinia* species; but differs in the presence of a DNA region within an end of the element, known as RB-HPI_ECOR31_ (right border-high pathogenicity island ECOR31) (see Fig. 1B, ICEs from *E. coli* ECOR31 and *Y. pestis* KIM) [43]. This region includes a pMET1-related T4SS, which made HPI_ECOR31_ the first pathogenicity island to be found harboring a complete set of conjugative plasmid genes [43]. Furthermore, the complete HPI_ECOR31_ can be excised from the chromosome, circularized, and transferred indicating that it is an integrative conjugative element (ICE) [43], [53]. These properties taken together with the structure of high-pathogenicity islands found in *Yersinia* species and other *Enterobacteriaceae* led to the suggestion that HPI_ECOR31_ is their progenitor [43]. It is possible that this is also the origin of the T4SS found in pCRY and pMET1, or conversely it has been captured from a plasmid related to pCRY or pMET1. These findings demonstrate the high rate of genetic exchanges that occurs between *Yersinia* species and other *Enterobacteriaceae*. These considerations also speak in favor of a high risk of acquisition of antibiotic resistance or virulence traits by pathogenic *Yersinia* species.

## Materials and Methods

### Bacterial strains and plasmids

Plasmid pMET1 was originally isolated from *K. pneumoniae* FC1, a strain isolated in a pediatric unit in Mendoza, Argentina (Hospital Luis C. Lagomaggiore) [12]. Plasmid pMET1 DNA was introduced into *Escherichia coli* XL1Blue (*recA1 endA1 gyrA96 thi-1 hsdR17 supE44 relA1 lac* [F′ *proAB lacI* ^q^ *Z*M15 Tn*10*]) (Stratagene) by transformation for purification and further manipulations. Recombinant clones including fragments of pMET1 were hosted in *E. coli* XL1Blue or *E. coli* TOP10 F^−^ *mcrA* Δ(*mrr*-*hsdRMS-mcrBC*) ϕ80*lacZ*ΔM15 Δ*lacX74 recA1 araD139* Δ(*ara-leu*)7697 *galU galK rpsL* (*Str^R^*) *endA1 nupG* (Invitrogen). *E. coli* C2110 *polA gyrA* Rif^r^ [54] was used as host to isolate the pMET1 replication region. Recombinant clones were generated using pUC18 [55], pCR2.1 (Invitrogen), or pSMART (Lucigen) as a cloning vectors. Plasmid pUC4K [56] was the source of the kanamycin resistance fragment (*aph* gene).

### Bacterial growth medium and general procedures

Growth of bacteria was in Lennox L broth (1% tryptone, 0.5% yeast extract, 0.5% NaCl). Plasmid DNA was prepared using the Qiagen plasmid mini kit (Qiagen, Inc.). Recombinant clones were sequenced using BigDye (ABI) and DYEnamic ET (Amersham) chemistries on an ABI Prism (model 310 or 3100) instrument and the appropriate commercial or custom-designed primers. The complete circular nucleotide sequence of pMET1 has been deposited in the GenBank sequence library and assigned the accession number EU383016. Sequences were examined and assembled with Sequencher 4.7 software (Gene Codes Corp.). DNA and protein sequence analyses were performed using the CLUSTAL W program (Pôle Bio-Informatique Lyonnais server [http://pbil.ibcp.fr/cgi-bin/npsa_automat.plpagenpsa_clustalw.html]) [57], the Promoter prediction code (http://www.fruitfly.org/seq_tools/promoter.html) [58], the Basic local alignment search tool (BLAST) (http://www.ncbi.nlm.nih.gov/BLAST/) [59], and Artemis (http://www.sanger.ac.uk/Software/Artemis/). The sequence logo was generated using the Weblogo web based application (http://weblogo.berkeley.edu/) [60], [61].
